# Topological classification of dynamical quantum phase transitions in the xy chain

**DOI:** 10.1038/s41598-020-69621-8

**Published:** 2020-07-29

**Authors:** Sergio Porta, Fabio Cavaliere, Maura Sassetti, Niccolò Traverso Ziani

**Affiliations:** 1 Dipartimento di Fisica, Università di Genova, 16146 Genova, Italy; 2 SPIN-CNR, 16146 Genova, Italy

**Keywords:** Phase transitions and critical phenomena, Topological matter

## Abstract

Understanding the properties of far-from-equilibrium quantum systems is becoming a major challenge of both fundamental and applied physics. For instance, the lack of thermalization in integrable and (many body) localized systems provides new insights in the understanding of the relaxation dynamics of quantum phases. On a more applicative side, the possibility of exploiting the properties of far-from-equilibrium states, for example in pump-probe experiments, opens unprecedented scenarios. The effort in providing a classification of far-from-equilibrium phases, in terms of local or topological order parameters, is hence intense. In this context, the concept of Dynamical Quantum Phase Transition (DQPT) has been introduced. A DQPT is (roughly) defined as a zero of the Loschmidt-Echo as a function of time and represents a natural non-equilibrium counterpart of a thermal phase transition. Here, we investigate the DQPTs occurring in the quantum xy chain subject to a quantum quench of finite duration. We show that the number of distinct DQPTs can vary as the duration of the quantum quench is varied. However, the parity of such number only depends on the pre-quench and post-quench Hamiltonians and is related to a topological invariant.

## Introduction

The study of far-from-equilibrium quantum states is a fruitful and intense branch of the research in physics. The ultimate aim, from the point of view of applications, is to engineer quantum states on demand employing time-dependent external perturbations^1, 2^. In this context, it has been shown that periodic drivings can lead, for example, to topological phases (that are promising for spintronics and quantum computation purposes)^3–5^, can generate or enhance superconductivity^6–10^, and can result in new phases such as discrete time crystals^11–13^. From the fundamental side, besides the possibility of engineering new phases and access solid state counterparts of quantum optics, the advances in the control of trapped ions^14, 15^, ultracold gases^16^, nitrogen vacancy centres in diamonds^17, 18^, and in condensed matter environments, of pump probe experiments^19, 20^, enable to inspect the dynamics of isolated quantum systems and hence a better understanding of thermalization. In this context, depending on the nature of the system under investigation, many scenarios can take place. In the thermodynamic limit, most systems follow the eigenstate thermalization hypothesis^21–23^, that is, the expectation values of local observables are thermal. However, notable exceptions of both fundamental and technological interest exist.(Topological) Anderson^24–26^ and many body localized^27–29^ phases as well as integrable systems^30–32^ are characterized by extensive sets of local or quasi-local conserved quantities. In these cases, if quantum information is stored in the initial state, such information is still available locally at later times and hence the system does not thermalize. In localized systems, the lack of thermalization is robust with respect to the parameters of the model, but the statistical ensemble describing the long time behavior cannot be constructed a priori. In integrable systems, the lack of thermalization is a fine tuned property but the long time average value of local observables can be described by the so called Generalized Gibbs Ensemble (GGE). If integrability is only weakly broken, the GGE describes a long lived pre-thermal plateau^33–36^. The usual (gedanken) experiment envisioned to access the main thermalization properties just discussed is the following: The system is prepared in the ground state of a Hamiltonian *H*(*g*), where the dependence on a parameter *g* is made explicit. At some time $t_0$ the parameter *g* varies, suddenly or according to some specific finite duration protocol, to a new constant value $g'$. If the variation of the parameter is abrupt, this procedure is dubbed sudden quantum quench, otherwise it is called finite duration quantum quench. Aside from issues related to thermalization, that are mostly inspected by means of the long time behaviour of the systems, quantum quenches are also interesting at finite times. In translational invariant systems, two point correlators are characterized by a light-cone structure^37–41^. This intriguing phenomenon is related to the fact that information can only propagate at finite velocity. More recently, it was discovered that some systems undergoing a quantum quench can show a non-analytical behaviour as a function of time, in quantities related to the Loschmidt echo^42^ (see Fig. 3 and “Discussion”). Such non-analiticities, dubbed Dynamical Quantum Phase Transitions^43^ (DQPTs) bear a similarity with thermal phase transitions and represent an attempt to characterize far-from-equilibrium phases^44^. Remarkably, DQPTs can survive even in the presence of dissipative baths^45^. At first, the presence of DQPTs following a quantum quench appeared to be linked to the fact that the pre-quench and the post quench parameters of the Hamiltonian represented points, in a parameter space, that could only be connected by a line crossing a quantum critical line. It was however shown that this is not necessarily the case^46, 47^: for example, sudden quantum quenches in the quantum xy chain, can display DQPTs even when they take place between points in the parameter space belonging to the same quantum phase^47^. Conversely, quantum quenches between phases separated by quantum critical lines can show the absence of DQPTs. In this article, we extend the study of the xy chain to finite duration quantum quenches, which have been proven in several instances to be drastically different from their sudden counterpart^48–50^. We find that the number ${{\mathscr {N}}}$ of (inequivalent) quantum DQPT can indeed be tuned by means of the duration of the quantum quench. Moreover, we show that, surprisingly, the parity of ${{\mathscr {N}}}$ does not depend on the quench duration and is related to a topological invariant. A clarification is here in order: in the absence of dissipation the DQPTs periodically repeat infinitely many times. Throughout the manuscript, we will indicate with sentences like ’the system exhibits a single/multiple DQPT/DQPTs’ the occurrence of one/multiple periods.

To be more specific, we consider the xy chain in a transverse magnetic field with periodic boundary conditions^51^. Its Hamiltonian can be written as

1 $$\begin{aligned} H(t)=J\sum _{j=1}^{N}\left[ \left( \dfrac{1+\gamma (t)}{2}\right) \sigma ^x_j \sigma ^x_{j+1}+\left( \dfrac{1-\gamma (t)}{2}\right) \sigma ^y_j\sigma ^y_{j+1}-h(t) \sigma ^z_j\right] , \end{aligned}$$

where $\sigma ^i_j$ (with $i=x,y,z$) are the Pauli matrices which describe spin operators on the *j*-th lattice site of the spin chain, *N* is the number of sites, $\gamma (t)$ is the anisotropy parameter and *h*(*t*) is the external magnetic field. From now on we set $J=\hbar =1$. By means of a Jordan-Wigner transformation, this model can be mapped onto a chain of free fermions with superconductive correlations^51^. We will, for simplicity, concentrate on the case of an even number of fermions. The results are not qualitatively affected by this choice.

**Figure 1:**
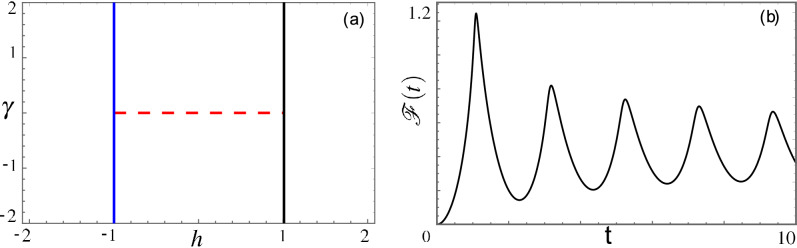
(**a**) Phase diagram of the xy model as a function of the anisotropy $\gamma$ and the external magnetic field *h*. The blue line represents the quantum critical line $h=-1$ and the black plain line the quantum critical line $h=1$. These two lines are in the Ising universality class. The red dashed line represents the $\gamma =0$ quantum critical segment, in the xx universality class. (**b**) The black solid line represents ${{\mathscr {F}}}(t)$ as a function of time in units $\hbar /J$ for a sudden quench. No kinks are present. Quench parameters: $(h_0,\gamma _0) = (2,2)$, $(h_1, \gamma _1) = (-2,2)$ (from initial to final points critical lines are crossed, see panel (**a**)).

In the static case, the model is characterized by a rich phase diagram^51^. As shown in Fig. 1a, three different Equilibrium Quantum Phase transitions (EQPTs) lines are present: one of them belongs to the universality class of the xx model (the quantum critical segment is $\gamma =0$, $|h|<1$) and the others are the phase transition lines of the 1D Quantum Ising model (the critical lines are given by $|h|=1$). The EQPTs are visible in the spectrum $\epsilon _q(\gamma ,h)$ as gap closings in the thermodynamic limit. In fact, one finds $\epsilon _q(\gamma ,h)=\sqrt{(h-\cos q)^2+\gamma ^2 \sin ^2 q}$, with $q=\frac{\pi }{N}(2n+1)$ labelling the quasi-momentum of the fermions.

Explicitly, the diagonalized Hamiltonian in terms of fermionic quasi-particles reads, in the momentum representation,

2 $$\begin{aligned} H=\dfrac{1}{2} \sum _{q=0}^{\pi } \Phi ^\dag _q \left( \begin{array}{cc} \epsilon _q(\gamma ,h) &{} 0 \\ 0 &{} -\epsilon _q(\gamma ,h) \end{array} \right) \Phi _q , \end{aligned}$$

where $\Phi ^\dag _q = \left( \chi _q^\dag , \chi _{-q}\right)$ and $\chi _q$ is the quasi-particle fermionic operator.

We will here assume that the parameters are constant up to $t=0$ and can afterwards vary in time. A sudden variation of the parameters has been extensively studied both in the unitary and in the dissipative framework^52–59^. Under these conditions DQPTs, a non-analytic behaviour of the quantity

3 $$\begin{aligned} {{\mathscr {F}}}(t)=-\lim \limits _{N\rightarrow \infty } \dfrac{1}{N} \ln {{\mathscr {G}}}(t). \end{aligned}$$

as a function of time *t* are an interesting object to study. Here, ${{\mathscr {G}}}(t)=|\langle 0|0(t)\rangle |$ is the Loschmidt overlap between the ground state $|0\rangle$ of the Hamiltonian for $t<0$ and the time evolved state $|0(t)\rangle$ at time *t*. The quantity ${{\mathscr {F}}}(t)$ bears similarities with the free energy, and hence its non-analyticities are to some extent analogous to phase transitions. Sudden quantum quenches of the form $(\gamma (t),h(t))=(\gamma _0\Theta (-t)+\gamma _1\Theta (t),h_0\Theta (-t)+h_1\Theta (t))$, where $\Theta (t)$ is the Heaviside step function, have been considered in connection to DQPTs in the xy model^47^. DQPTs can appear, as usual, when the sudden quench is performed across a quantum critical line. However, the xy chain has the peculiarity that DQPTs can appear even for sudden quantum quenches in which the initial and the final values for *h* and $\gamma$ can be connected in parameter space without crossing a quantum critical line. At the same time, when the quantum quench takes place between points in parameter space that cannot be connected without going through an equilibrium critical line, it can happen that DQPTs are absent. This very peculiar behavior is shown in Fig. 1b. To better understand this intriguing phenomenon and in order to deepen the characterization of the DQPTs, we inspect, in this article, their fate when the quantum quench is not sudden. What we will show is that, given the pre-quench and post-quench parameters it is not possible to a priori determine if DQPTs are present of not. However, the parity of the DQPTs can be stated irrespective of the quench protocol.

## Results

### Quantum quench of finite duration

We consider a time-dependent Hamiltonian of the form

4 $$\begin{aligned} H(t)=H(\gamma (t),h(t)) \end{aligned}$$

where the quench protocol is encoded in the explicit time evolution of the model parameters, given by

5 $$\begin{aligned} \gamma (t)= \,& {} \gamma _0+(\gamma _1-\gamma _0)Q_\gamma (t) , \end{aligned}$$

6 $$\begin{aligned} h(t)=\, & {} h_0+(h_1-h_0)Q_h(t) . \end{aligned}$$

The function $Q_l(t)$, with $l=\gamma ,h$, is determined by the quench duration $\tau$ and its functional dependence on time, which is left generic at this stage. In general, it is defined as

7 $$\begin{aligned} Q_l(t)= \left\{ \begin{array}{ll} 0 &{} t\le 0 \\ {\tilde{Q}}_l(t) &{} 0< t<\tau \\ 1 &{} t\ge \tau \end{array} \right.. \end{aligned}$$

Note that, while we here specifically address the Hamiltonian in Eq. (1), the analysis we provide is rather general when dealing with $2\times 2$ Hamiltonians in the Bogoliubov-De Gennes form. The latter, in general, allows to diagonalize mean–field superconducting Hamiltonians with *n* bands employing the same techniques used for non-superconducting fermionic systems with 2*n* bands^60^. The true spectrum of the Hamiltonian is then usually recovered by taking the positive energy eigenvalues, since an artificial particle-hole symmetry is introduced during the procedure. The time evolution of the fermionic operators diagonalizing the initial Hamiltonian $H(\gamma _0,h_0)$ can be formally obtained by means of the ansatz

8 $$\begin{aligned} \Phi _q^0(t)=\left[ \begin{array}{c} \chi _q^0(t) \\ \chi _{-q}^{0\dag }(t) \end{array} \right] = \left[ \begin{array}{cc} f_{q,1}(t) &{} g_{q,1}(t) \\ f_{q,2}(t) &{} g_{q,2}(t) \end{array} \right] \left[ \begin{array}{c} \chi _q^0 \\ \chi _{-q}^{0\dag } \end{array} \right] = M_q(t)\Phi _q^0 , \end{aligned}$$

where the time dependence is transferred to the coefficients $f_{q,m}$ and $g_{q,m}$, with $m=1,2$. The initial conditions are, for quenching from the ground state, ${f_{q,1}(0)=g_{q,2}(0)=1}$ and ${f_{q,2}(0)=g_{q,1}(0)=0}$. To ensure the validity of the anticommutation relations during the dynamics, the coefficients have to satisfy, $\forall t$, the condition ${|f_{q,m}(t)|^2+|g_{q,m}(t)|^2=1}$. The Heisenberg equation for the fermionic operators gives two coupled systems of differential equations for the time-dependent quantities. Defining here implicitly the two component objects $F_q(t)$ and $G_q(t)$, we get

9 $$\begin{aligned} \frac{dF_q(t)}{dt}= & {} \frac{d}{dt}\left[ \begin{array}{c} f_{q,1}(t) \\ f_{q,2}(t) \end{array} \right] =\dfrac{1}{2i} \left[ \begin{array}{cc} a_{q}(t) &{} b_{q}(t) \\ b_{q}(t) &{} -a_{q}(t) \end{array} \right] \left[ \begin{array}{c} f_{q,1}(t) \\ f_{q,2}(t) \end{array} \right] = {{\mathscr {M}}}_q(t)F_q , \end{aligned}$$

10 $$\begin{aligned} \frac{dG_q(t)}{dt}= & {} \frac{d}{dt}\left[ \begin{array}{c} g_{q,1}(t) \\ g_{q,2}(t) \end{array} \right] = \dfrac{1}{2i}\left[ \begin{array}{cc} a_{q}(t) &{} b_{q}(t) \\ b_{q}(t) &{} -a_{q}(t) \end{array} \right] \left[ \begin{array}{c} g_{q,1}(t) \\ g_{q,2}(t) \end{array} \right] = {{\mathscr {M}}}_q(t)G_q . \end{aligned}$$

where

11 $$\begin{aligned} a_{q}(t)= & {} \left[ h(t)-\cos q\right] \cos 2\theta _q(\gamma _0,h_0)+\gamma (t)\sin q\sin 2\theta _q(\gamma _0,h_0) , \end{aligned}$$

12 $$\begin{aligned} b_{q}(t)= & {} \left[ h(t)-\cos q\right] \sin 2\theta _q((\gamma _0,h_0))-\gamma (t)\sin q\cos 2\theta _q(\gamma _0,h_0) . \end{aligned}$$

Here, $\theta _q(\gamma ,h)$ is defined via $\tan \left[ 2\theta _q(\gamma ,h)\right] = \gamma \sin \left[ q/(h-\cos q)\right]$.

We note that, in general, the systems cannot be solved analytically during the quench, while it is easy to obtain the post-quench solution by matching conditions for $F_q(t)$ and $G_q(t)$ in $t=\tau$.

In the post-quench regime, the coefficients $a_{q}(t\ge \tau )=a_{q}(\tau )$ and $b_{q}(t\ge \tau )=b_{q}(\tau )$ are constant. In this regime, we obtain

13 $$\begin{aligned} f_{q,1}(t\ge \tau )=\, & {} f_{q,1}(\tau )\cos [\epsilon _q(\gamma _1,h_1) (t-\tau )/2] \nonumber \\&-i \left[ f_{q,1}(\tau )\cos 2\Theta _q-f_{q,2}(\tau )\sin 2\Theta _q\right] \sin [\epsilon _q(\gamma _1,h_1) (t-\tau )/2] , \end{aligned}$$

14 $$\begin{aligned} f_{q,2}(t\ge \tau )=\, & {} f_{q,2}(\tau )\cos [\epsilon _q(\gamma _1,h_1) (t-\tau )/2] \nonumber \\&+i \left[ f_{q,2}(\tau )\cos 2\Theta _q+f_{q,1}(\tau )\sin 2\Theta _q\right] \sin [\epsilon _q(\gamma _1,\theta _1) (t-\tau )/2] , \end{aligned}$$

where $\Theta _q=\theta _q(\gamma _1,h_1)-\theta _q(\gamma _0,h_0)$. The solution for $G_q(t)$ can be obtained from $F_q$ since $f_{q,1}(t)=g_{q,2}^*(t)$ and $f_{q,2}(t)=-g_{q,1}^*(t)$. The full information on the quench protocol is encoded in $f_{q,1/2}(\tau )$.

We are now able to evaluate the expectation values of the occupation numbers related to the fermionic operators $\chi _q^1, \chi _{-q}^{1\dag }$, which diagonalize the final Hamiltonian $H(\gamma _1,h_1)$, over the initial state $| 0 \rangle$. We label the eigenvalues of $H(\gamma _1,h_1)$ as $\epsilon ^1_q$ Such occupation numbers are conserved quantities in the post-quench regime and hence represent the steady state occupations. They are hence labelled with the index *GGE*. We obtain

15 $$\begin{aligned} N_q^{{\text {GGE}}}=\, & {} \langle 0|\chi _q^{1\dag }\chi _q^{1}|0 \rangle = \langle 0|\chi _{-q}^{1\dag }\chi _{-q}^{1}|0 \rangle \nonumber \\=\, & {} |f_{q,1}(\tau )|^2 \sin ^2\Theta _q + |f_{q,2}(\tau )|^2 \cos ^2\Theta _q+ 2{\text {Re}}\left[ f_{q,1}(\tau )f_{q,2}^*(\tau )\right] \cos \Theta _q \sin \Theta _q \end{aligned}$$

where $| 0 \rangle$, as mentioned before, represents the ground state of the initial Hamiltonian $H(\gamma _0,h_0)$ and is defined as the vacuum of quasi-particles, i.e. $\chi _q^0 (t<0)| 0 \rangle =0$, for every *q*. Crucially for the following, $N_{0/\pi }^{{\text {GGE}}}$ do not depend on the quench protocol. This is essentially due to the fact that the Fourier-transformed Hamiltonian is diagonal in terms of the Jordan-Wigner fermions at $k=0/\pi$^51^.

### Loschmidt overlap

In this section we evaluate the Loschmidt overlap ${{\mathscr {G}}}(t)$ following a non sudden quantum quench. Thanks to the solution to the full dynamics of the fermionic operators previously introduced, it can be shown that, in the thermodynamic limit,

16 $$\begin{aligned} {{\mathscr {F}}}(t)=-\dfrac{1}{\pi }\int _{0}^{\pi } dq \ln |f_{q,1}(t)| , \end{aligned}$$

where in the post-quench regime Eq. 16 can be made explicit by means of Eq. 13 to obtain

17 $$\begin{aligned} {{\mathscr {F}}}(t\ge \tau )=-\dfrac{1}{\pi }\int _{0}^{\pi } dq \ln |f_{q,1}(\tau )\cos [\epsilon _q^1 (t-\tau )/2]-i \left[ f_{q,1}(\tau )\cos 2\Theta _q-f_{q,2}(\tau )\sin 2\Theta _q\right] \sin [\epsilon _q^1 (t-\tau )/2]|. \end{aligned}$$

In order to evaluate analytically the zeros of the argument of the log-function we rewrite $f_{q,1}(t\ge \tau )$ in the form

18 $$\begin{aligned} f_{q,1}(t\ge \tau )=\cos \left[ \epsilon _q^1 (t-\tau )/2-\varphi _q\right] , \end{aligned}$$

where

19 $$\begin{aligned} \varphi _q= \arctan \left[ -i (f_{q,1}(\tau ) \cos 2\Theta _q - f_{q,2}(\tau ) \sin 2\Theta _q )\right] . \end{aligned}$$

Since $\varphi _q$ is a complex function, we now have to find the momenta $q^*_i$ such that its imaginary part is vanishing. Once the imaginary part is set to zero, the condition is transferred to the real part. However, the real part evolves in time harmonically when the imaginary part is zero, so that one can find the times corresponding DQPTs. It is hence the quantity Im($\varphi _q$), that strongly depends on the quench protocol, that sets the existence and the number of non equivalent DQPTs. The times where the dynamical free energy shows a non-analytic behavior are given by

20 $$\begin{aligned} t_{i,n}^*=\tau +\left[ (2n+1)\pi +2\varphi _{q_i^*}\right] /\epsilon _{q_i^*}^1 . \end{aligned}$$

**Figure 2:**
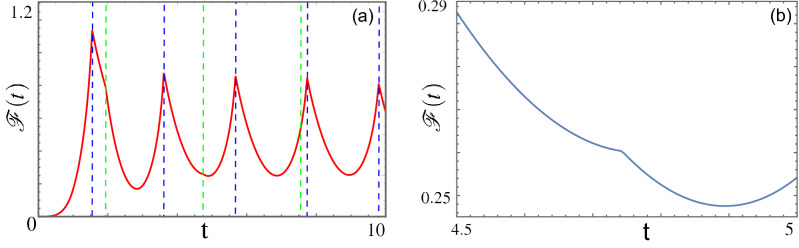
Plot of the function ${{\mathscr {F}}}(t)$ as a function of time for $\tau =1$. The vertical lines in (**a**) indicate the DQPTs. (**b**) A zoom of (**a**), around the second green line. Other parameters as in Fig. 1b: $(h_0,\gamma _0) = (2,2)$, $(h_1, \gamma _1) = (-2,2)$.

Interestingly, the number of $q^*_i$, can vary as a function of the initial and final phases as well as the quench duration and protocol. As a striking example, see Fig. 2. Here, it is shown that for non-sudden quantum quenches DQPTs exist even though the corresponding sudden quench does not show any (see Fig. 1b). Both the blue and the green lines in panel (a) highlight DQPTs, although the non-analytical behavior corresponding to the green lines is weaker. A zoom of the latter is shown in panel (b).

**Figure 3:**
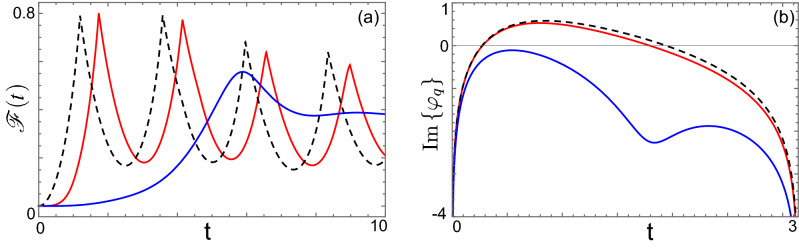
(**a**) Plot of ${{\mathscr {F}}}(t)$ as a function of time for $\tau =0$, (black dashed), $\tau =1$ (red) and $\tau =10$ (blue). (**b**) Plot of ${\text {Im}}\{\varphi _q\}$ as a function of the momentum *q* for different values of the quench duration: $\tau = 0$ (black dashed), $\tau = 1$ (red), $\tau = 5$ (blue).

Furthermore, every zero gives rise to a new non-equilibrium time scale in the dynamics. The converse is also possible. Fig. 3 shows a scenario in which DQPTs are present in the sudden quench case, but disappear when the ramp is sufficiently slow.

**Figure 4:**
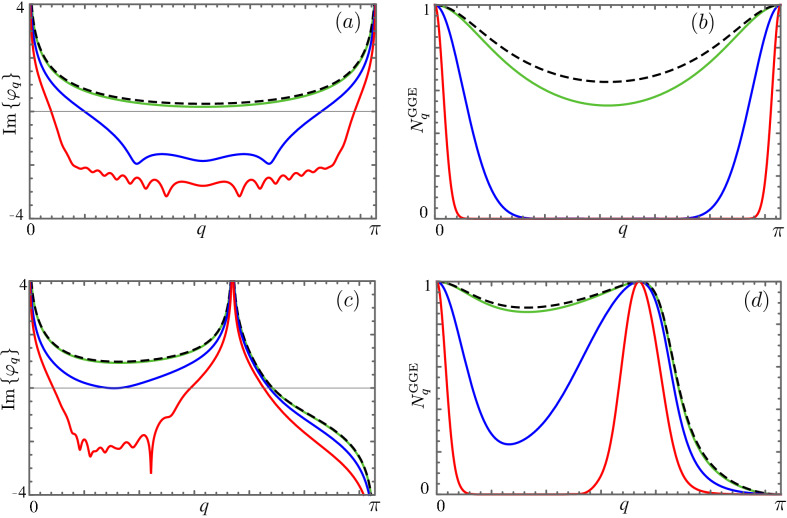
Plot of ${\text {Im}}\{\varphi _q\}$ (left panels) and $N^{GGE}_q$ (right panel) as a function of the momentum *q* for different values of the quench duration: $\tau = 0$ (black dashed), $\tau = 1$ (green), $\tau = 10$ (blue) and $\tau = 100$ (red). Quench parameters: Top figures $(h_0, \gamma _0) = (2,1.5)$, $(h_1, \gamma _1) = (-2,1.5)$, Bottom figures $(h_0, \gamma _0) = (2,-2)$, $(h_1, \gamma _1) = (-0.5,0.2)$.

Interestingly, it can be inferred from the general discussion that will be presented in the following, that the sign of the quantities Im($\varphi _{0}$) and Im($\varphi _{\pi }$) only depends on the initial and the final values of the parameters. See Fig. 4a,c. Moreover, at these two special points of the Brillouin zone, $\varphi _q$ can be expressed as a simple function of the occupation numbers of the post-quench fermionic excitations as

21 $$\begin{aligned} \varphi _{0/\pi }=-i \,{\text {arctanh}}[1-2 N^{GGE}_{0/\pi }], \end{aligned}$$

where $N^{GGE}_{0/\pi }$ is given in Eq. (15) and, therefore, does not depend on the quench protocol.

In this respect, we are hence able to simply evaluate the sign of the function Im($\varphi _q$) at the edges of the region we are inspecting. In particular, it is straightforward to show, from Eq. (21), that every time the occupation numbers $N^{GGE}_{0/\pi }$ jump from 0 to 1 or viceversa, the sign of Im($\varphi _{\pi /0}$) changes accordingly. This relation is apparent in Fig. 4, where some prototypical examples of both these functions are plotted.

However, the peculiar behavior of the occupation numbers and, consequently, of the sign of the imaginary part of $\varphi _q$, is strictly related to the xy model phase diagram, illustrated in the “Introduction” and in particular in Fig. 1a. Indeed, there is a well-defined correspondence between the phases connected by the quench, or rather the critical lines involved, and the value of the occupation numbers $N^{GGE}_q$ in $q=0,\pi$.

The value of $N^{GGE}_\pi$ is vanishing if the quench starts and finishes in the same phase, while it jumps to 1 when the $h=-1$ critical line separates the initial and final phases connected by the quench. On the other hand, but analogously, $N^{GGE}_0$ have the same jump when the $h=1$ critical line is involved. The mentioned feature can be analytically shown by inspecting Eq. (15) in the particular cases outlined. The number of zeros of Im($\varphi _q$) can hence be addressed. Nevertheless, the argument is not straightforward since a third independent non-analytical behavior as a function of the quench parameters can occur, see Fig. 4c. Going back to the phase diagram of the model, we note that the horizontal critical line $\gamma =0$ has not yet been considered. Such equilibrium critical line may give rise, in particular circumstances, to an additional infinity with respect to the ones in $q=0/\pi$ in Im($\varphi _{{\bar{q}}}$), with ${\bar{q}}=\arccos \left( \dfrac{h_0\gamma _1-h_1\gamma _0}{\gamma _1-\gamma _0}\right)$, i.e. if ${\bar{q}}\in {\mathbb {R}}$. However, given the continuity of the occupation numbers $N^{GGE}_q$ and the relation

22 $$\begin{aligned} {\text {Im}}(\varphi _q) \sim -{\text {arctanh}}[1-2 N^{GGE}_{q}] \end{aligned}$$

in the proximity of $q=0,\pi ,{{\bar{q}}}$, we observe that Im($\varphi _q$) does not change sign in the proximity of these non-analytical points. We can conclude, therefore, that the number of its zeros, irrespective of what happens in $q={\bar{q}}$, is even(odd) if ${\text {Sgn}} [{\text {Im}}(\varphi _0)] \cdot {\text {Sgn}} [{\text {Im}}(\varphi _\pi )]$ is even (odd). If we transpose the argument on the occupation numbers, we obtain that, analogously, the number of zeros of Im($\varphi _q$) is even if $N^{GGE,s}_0 = N^{GGE,s}_\pi$ and odd if $N^{GGE,s}_0 \ne N^{GGE,s}_\pi$. Examples are shown in Fig. 4. In panel (a) it is shown that when the quench is sudden there are no DQPTs despite the fact that the initial and the final Hamiltonians are necessarily separated by critical lines. For non-sudden quench protocols, on the other hand, DQPTs emerge. The correspondent behavior of the occupation numbers is shown in panel (b). In panel (c) the scenario in which the number of DQPTs switches from one to three upon varying the quench duration is shown. In analogy to panel (b), panel (d) shows the corresponding behavior of the occupation numbers.

Note that, importantly, the usual paradigm stating that the DQPTs occur whenever the occupation number of the quasi-particle states crosses 1/2 is here violated, as also observed for non-sudden quantum quenches in the Ising model^48^. Even more significant is the fact that despite the number of zeros in Im($\varphi _q$) can vary as the quantum quench duration is changed, as already discussed for a generalized xy-like spin model^49^, the parity of such number only depends on the initial and final parameters. With respect to DQPTs, the quantum quenches in the xy chain in a transverse field can hence be classified on the basis of the parity of DQPTs they induce. Note that this classification allows us to make a distinction between quantum quenches that can show or not DQPTs depending on the quantum quench duration and those that are bounded to show DQPTs. According to the initial and final parameters one can hence have ’robust’ (odd parity) or ’non-robust’ (even parity) DQPTs. This is the main result of this article. In the next subsection we exhibit a topological invariant that allows us to put this ${\mathbb {Z}}_2$ classification on a more formal basis.

### Dynamical topological invariant

Following Yang’s work^61^, we first introduce a Dynamical Topological Invariant for the case of a sudden quantum quench. The possibility to extend Ref.^61^, which deals with non-superconducting systems with two bands, to the case of a superconducting model with just one band—such as the one describing the Wigner-Jordan fermions—is ensured by the Bogoliubov-de Gennes 2X2 representation of the Hamiltonian, in the particle-hole space. We then generalize it to the finite-duration quench case and draw a connection to the parity of the DQPTs in the xy chain in a transverse magnetic field.

We start by considering a generic 1D two band Hamiltonian with (discrete) translational symmetry. In the momentum representation, the Hamiltonian can be represented as the sum of two level systems and, *k* by *k*, it reads

23 $$\begin{aligned} h(k,g) =\vec {d}(k,g)\cdot \vec {\sigma } \end{aligned}$$

where *k* is an index for the (quasi-)momentum, *g* represents a set of parameters appearing in the Hamiltonian (for example $g=(\gamma ,h)$ for the xy model), $\vec {d}(k,g)$ is a three-vector and $\vec {\sigma }$ is the vector containing the Pauli matrices. Terms proportional to the identity matrix are not relevant in the following and hence are not considered. The diagonal elements of density matrix are given by

24 $$\begin{aligned} \rho _\pm (k,g) = | \psi _\pm (k,g) \rangle \langle \psi _\pm (k,g) |=\frac{1}{2} \left[ 1\pm {\hat{d}}(k,g)\cdot \vec {\sigma }\right] , \end{aligned}$$

where $| \psi _\pm (k,g) \rangle$ are the Hamiltonian eigenvectors, with $| \psi _-(k,g) \rangle$ lower in energy, and ${\hat{d}}(k,g)= \vec {d}(k,g)/|\vec {d}(k,g)|$.

We now consider a sudden quench transforming the initial Hamiltonian $h(k,g_0)$ into $h(k,g_1)$, and we study the non-equilibrium properties of the system if it is prepared in the ground state of $h(k,g_0)$. By means of the Liouville-Von Neumann equation, we obtain the time evolution of the density matrix component of the occupied band. Explicitly we have

25 $$\begin{aligned} \rho _-(k,g_1,t)=\frac{1}{2} \left[ 1-\tilde{d}(k,g_0,g_1,t)\cdot \vec {\sigma }\right] , \end{aligned}$$

where

26 $$\begin{aligned} \tilde{d}(k,g_0,g_1,t)= \,& {} {\hat{d}}(k,g_0)\cos \left[ 2|\vec {d}(k,g_1)|t\right] +{\hat{d}}(k,g_1)\left[ {\hat{d}}(k,g_0)\cdot {\hat{d}}(k,g_1)\right] \sin ^2\left[ |\vec {d}(k,g_1)|t\right] \nonumber \\&+{\hat{d}}(k,g_0)\times {\hat{d}}(k,g_1)\sin \left[ 2|\vec {d}(k,g_1)|t\right] . \end{aligned}$$

Note that $\tilde{d}(k,g_0,g_1,t)$ is a periodic function both of time, with periodicity $\pi /|\vec {d}(g_1,k)|$, and of momentum, with periodicity $2\pi$. This means that the topology of the Brillouin zone, as well as of time, is $S^1$ so that the momentum-time manifold has, in general, a topology $T^2$. However, it can happen for some points ${k_m}$ in the BZ (called here fixed points), that the vector ${\tilde{d}}(k,g_0,g_1,t)$ does not evolve in time. This translates to a momentum-time manifold that becomes a set of spheres $S^2$, the number of which equals the number of fixed points. It is hence possible to define the Chern numbers

27 $$\begin{aligned} C^m_{\text {dyn}}=\dfrac{1}{4\pi } \int _{k_m}^{k_{m+1}} dk\int _{0}^{\pi }dt' \left( \tilde{d}(k,g_0,g_1,t)\times \partial _{t'}\tilde{d}(k,g_0,g_1,t)\right) \cdot \partial _k\tilde{d}(k,g_0,g_1,t) , \end{aligned}$$

where the time has been rescaled to $t'=t/|\vec {d}(k,g_1)|$. It can be shown that the numbers $C^m_{\text {dyn}}$ are integers^61^: they indicate if the mapping from the corresponding momentum-time sub-manifold to the Bloch vector is trivial ($C^m_{\text {dyn}}=0$) or not ($C^m_{\text {dyn}}=\pm 1$).

Going back to the sudden quantum quenches in the xy model, after recasting the Hamiltonian into the form of Eq. (23), one finds

28 $$\begin{aligned} C^m_{\text {dyn}}=\frac{\cos (2\Theta _{k_m})-\cos (2\Theta _{k_{m+1}})}{2}. \end{aligned}$$

Moreover, $k=0$ and $k=\pi$ are always fixed points and, at every fixed point, $\cos (2\Theta _{k_j})=\pm 1$.

Of particular interest is the quantity

29 $$\begin{aligned} C_{{\text {dyn}}}=\sum _{k_m \in [0,\pi ]} C_{{\text {dyn}}}^m = \dfrac{1}{2}\left( \cos \left[ 2\Theta _{0}\right] -\cos \left[ 2\Theta _{\pi }\right] \right) = N^{GGE,s}_\pi -N^{GGE,s}_0. \end{aligned}$$

In fact one finds $C_{{\text {dyn}}}=0$ if the population of the quasiparticles in $k=0$ is equal to the population at $k=\pi$, while we have $C_{{\text {dyn}}}=\pm 1$ if the populations are different.

Note that the last equality in Eq. (29) implies that $C_{{\text {dyn}}}$ is an integer. This fact draws an interesting link between the robustness of the DQPTs with respect to the duration of the quench and the topological number associated to the sudden quenches.

However, with respect to the topological indexes, the scenario in the case of non sudden quantum quenches is in principle rather different. In Eq. (26), instead of the unit vector ${\hat{d}}(k,g_0)$, the evolution for $t>\tau$ is regulated by the unit vector $\hat{d'}(k,g_0,\tau )$ describing the quantum state at time $\tau$. Since however the unit vector $\hat{d'}(k,g_0,\tau )$ cannot, in general, be found analytically, universal statements become a priori unlikely. Nevertheless, the fixed points can be obtained by studying the transformation which connects the two diagonalizing basis of the initial ($H_0$) and final ($H_1$) Hamiltonians, namely

30 $$\begin{aligned} \Phi _q^1= \left[ \begin{array}{c} \chi _q^1 \\ \chi _{-q}^{1\dag } \end{array} \right] = \left[ \begin{array}{rr} \cos \Theta _q &{} -\sin \Theta _q \\ \sin \Theta _q &{} \cos \Theta _q \end{array} \right] \left[ \begin{array}{c} \chi _q^0 \\ \chi _{-q}^{0\dag } \end{array} \right] = {{\mathscr {B}}}_q\Phi _q^0 \ . \end{aligned}$$

The transformation matrix ${{\mathscr {B}}}_q$, evaluated in the fixed point $q=k_m$, is the identity matrix if the corresponding critical line is crossed an even number of times, while it is $i\sigma ^y$ otherwise. Crucially, as previously discussed, $k=0$ and $k=\pi$ are fixed points even in the case of finite quantum quenches. This behaviour is due to the fact that one obtains the following differential equation for the vector ${{\hat{d}}}$ at the center and at the edge of the BZ,

31 $$\begin{aligned} \partial _t {\hat{d}}(0/\pi ,t)=2 \left[ 0,0,a_{0/\pi }(t)\right] \times {\hat{d}}(0/\pi ,t), \end{aligned}$$

with $a_q(t)$ given in Eq. (11) and the initial condition given by ${\hat{d}}(0/\pi ,t=0)=[0,0,a_{0/\pi }(t=0)]$. Hence, explicitly, ${\hat{d}}(k,t)={\hat{d}}(k,0)$ for $0<t<\tau$ and $k=0,\pi$, so that these points are not affected by the quench protocol or its duration. The time evolution, once the final Hamiltonian has been reached, is hence given by Eq. 26, as in the sudden quench case. The sum of the dynamical topological invariants $C_{\text {dyn}}$ in half of the BZ is then independent of the quench duration, even if the single $C_{\text {dyn}}^m$ are not. In particular, whenever $C_{\text {dyn}}=0$, DQPTs are not robust and can thus be cancelled out (created) increasing the quench duration if in the sudden quench regime they are present (absent). When $C_{\text {dyn}}=\pm 1$ on the other hand, by varying the quench duration it is not possible to find a quench protocol where DQPTs disappear. So, the number of points in the momentum space which give rise to non-analyticities (see Eq. 20) can vary as a function of the quench duration and protocol, but its parity is determined solely by the initial and final Hamiltonian and coincides with $C_{\text {dyn}}$.

## Discussion

The results presented demonstrate that, within a model as simple as the xy chain in a transverse field, the effect of the time duration of non-sudden quantum quenches can be dramatic. In fact, the number and the very existence of DQPTs strongly depend on the quench duration. The drastic difference between the sudden and the non-sudden quench is shown in Fig. 2, where DQPTs are absent in the sudden quench case but emerge as the ramp duration is increased, and in Fig. 3, where the opposite scenario is represented: DQPTs are present in the sudden quench case and disappear for large $\tau$. However, surprisingly, the parity of the DQPTs is fixed once the initial and final parameters are fixed. We have associated this behaviour to a new topological invariant that essentially counts the parity of the number of *k*-states that evolve trivially in time after the quantum quench. The results we obtained ultimately rely on the fact that the post-quench occupation numbers of the states at $k=0$ and $k=\pi$ do not depend on the quench protocol. We stress that our results are not peculiar of quantum quenches from the ground state: as an example a non-sudden quench corresponds to a sudden quench from the state that the system has reached at the end of the protocol. Since we have shown that the ramp does not influence the occupations at $k=0$ and $k=\pi$, then the ${\mathbb {Z}}_2$ classification of the DQPTs applies to all pre-quench states that can be generated by unitarily time evolving the ground state. We can hence argue that this in general holds true for any initial state unitarily generated from the ground state. Moreover, the mechanism we discovered is not restricted to the xy chain as well: all the two-band (even in the BdG sense) one dimensional models with inversion symmetry show this phenomenology. In fact, since inversion symmetry is a discrete symmetry, and since $k=0$ and $k=\pi$ are inversion symmetric points, the populations at $k=0$ and $k=\pi$ are bound to be either one or zero at any time if the initial state is an Eigenstate of the inversion operator. For future investigations, it will be interesting to understand if, in models with broken inversion symmetry, even the ${\mathbb {Z}}_2$ classification is lost. More generally, our work stresses the experimentally relevant fact that DQPTs can be very sensitive to the quench protocol, both in a constructive and in a destructive way.
